# Alum Injection for Dysentery

**Published:** 1890-03-01

**Authors:** 


### ALUM INJECTION FOR DYSENTERY.

Dep.-Insp.-General H. Norbury, C.B., Naval Medical Department, contributes a case of dysentery cured by alum enemata (*Lancet*). The patient, a seaman, suffered from chronic dysentery, which, as usual in such cases, appears to have become at times sub-acute. "He had frequently suffered from tormina, had three to ten loose offensive stools daily accompanied by much tenesmus, and by occasional passage of blood, and he had become feeble and emaciated." He was carefully dieted, took compound ipecacuanha powders, aromatic sulphuric acid, decoction of hœmatoxylon, lead pill with opium, compound kino powder &c. &c., from which he derived no benefit. An enema containing half an-ounce of alum in ten ounces of water was prescribed, to be administered night and morning. It produced slight sweating at first, but the effect in a couple of days was remarkable, the stools were reduced to one or two daily, and shortly became of natural consistence. After eighteen days of this treatment the patient looked robust, and in a few days afterwards resumed his usual avocations. Now Dr. Norbury informs us the sailor contracted his dysentery abroad, and it may be inferred, although Dr. Norbury does not say so, that when the patient came under Dr. Norbury's care he had recently returned from abroad. We have so frequently known injections of various kinds fail in benefiting chronic dysentery, that we feel rather disposed to attribute recovery in this instance to other agencies than the alum, to which it is credited.

